# Nonlinear dynamic analysis of the pure “buckling” mechanism during blow-out trauma of the human orbit

**DOI:** 10.1038/s41598-020-72186-1

**Published:** 2020-09-17

**Authors:** Marcin Adam Zmuda Trzebiatowski, Paweł Kłosowski, Andrzej Skorek, Krzysztof Żerdzicki, Paweł Lemski, Mateusz Koberda

**Affiliations:** 1 Department of Structural Mechanics, Faculty of Civil and Environmental Engineering, Gdańsk University of Technology, 11/12 Gabriela Narutowicza St., 80-233 Gdańsk, Poland; 2 Department of Otolaryngology, Medical University of Gdańsk, 17 Mariana Smoluchowskiego St., Gdańsk, 80-214 Poland; 3 Department of Ophthalmology, Medical University of Gdańsk, 17 Mariana Smoluchowskiego St., Gdańsk, 80-214 Poland

**Keywords:** Computational models, Biomedical engineering, Bone

## Abstract

Considering the interplay between orbital bones and intraorbital soft tissues, commonly accepted patterns of the blow-out type of trauma within the human orbit require more thorough investigation to assess the minimal health-threatening impact value. Two different three-dimensional finite element method (FEM) models of the human orbital region were developed to simulate the pure “buckling” mechanism of orbital wall fracture in two variants: the model of orbital bone elements and the model of orbital bone, orbit and intraorbital tissue elements. The mechanical properties of the so-defined numerical skull fragment were applied to the model according to the unique laboratory tensile stress tests performed on small and fragile specimens of orbital bones as well as using the data available in the literature. The nonlinear transient analysis of the contact problem between bodies that differ substantially in terms of the Young’s modulus was carried out to investigate the interaction of different bodies within an instant injury. Potential damage areas were found within the lower orbital wall as well as the destructive load values for both FEM skull models (7,660 N and 8,520 N). Moreover, numerical simulations were validated by comparing them with computed tomography scans of real injuries.

## Introduction

Regardless of the time that has passed, patients suffering from blow-out fracture of an orbital wall are still considered to experience a serious diagnosis and therapeutic problem^1,2^. Such trauma requires strict cooperation between physicians representing a variety of different specializations, such as otolaryngologists, ophthalmologists, neurosurgeons, and maxillofacial and plastic surgeons^3^. Blow-out fracture affects the inferior as well as the medial wall, where the bony part of the orbit is the most fragile. Moreover, fractured sites often facilitate the herniation of intraorbital bodies, so they may become constrained by the rim of the fractured area as well as the trapdoor displacement of a thin orbital bone segment^4^. The direct consequence of blow-out trauma may cause additional symptoms, including diplopia, enophthalmos (the posterior displacement of the eyeball), loss of sensation over the upper lip and gums on the injured side, a handicapped upward gaze (as a result of entrapment of the inferior rectus muscle) or crepitus by palpation around the inferior orbital ridge^5^. There are also several potential factors that are responsible for the occurrence of blow-out trauma, such as competitive sports or military conflicts, but the most common are traffic accident cases and violent assaults, according to surveys and statistics from across various developed countries^6–8^.

The mechanism of orbital floor fracture via blow-out trauma was first described by Converse and Smith^9^. Afterwards, the theory was developed, and the mechanisms of blow-out fractures were systematized and described as two general mechanisms, namely, “hydraulic” and “buckling” mechanisms and are widely accepted today^10–12^. The “hydraulic” mechanism is the indirect one, where the striking force is applied at the eyeball causing the intraorbital hydraulic pressure to affect the surrounding bony orbit. However, the “buckling” mechanism (which has nothing to do with mechanical “buckling”) is the direct one, where the impact is being applied solely at the orbital rim causing the deflection of the thin and fragile lower wall of the orbit, while the orbital rim remains intact. In the experimental work of Fujino, dried human skulls were impacted by a brass striker with a mass of 0.42 kg equipped with a flat silicone rubber plate at the lower part of the striker^13^. The entire impact was applied at the infraorbital margin, and the “buckling” mechanism of the blow-out trauma was empirically indicated. Furthermore, a few years later, Fujino and Makino^14^ identified the “buckling” mechanism as the main factor of the blow-out fracture of a human orbit based on an evaluation of over 100 isolated blow-out clinical cases.

A few studies have investigated the biomechanics of blow-out fractures within the pure “buckling” mechanism. Nagasao et al.^15^ presented a study on the pure “buckling” mechanism within the shell FEM model based on real human skull geometry. Similar works were presented around the same time, e.g., the work of Al-Sukhun et al.^16^, nevertheless, those models were built upon an idealized geometry of the orbit. Similar investigations of circumorbital mechanics were also performed on skull models of other nonhuman primates, e.g., that by Ross et al.^17^. Only further works by Al Sukhun et al. as well as Patel et al. and Foletti et al. did not omit the intraorbital soft tissues during the numerical analysis of blow-out fracture^18–21^.

The present research is a continuation of the previous works of authors aiming to develop and possibly extend the issues raised by other authors previously involved in the abovementioned subject^22–24^. This research is aimed both at characterizing the mechanical properties of the orbit and investigating the role of soft tissue in modeling the “buckling” mechanism of human orbital fracture by constructing two different FEM models: a model of orbital bone elements (MOBE) and a model of orbital bone, orbit and intraorbital tissue elements (MOBOSE). In particular, both the stress and displacement distribution within the human orbit are analyzed, considering the effect of intraorbital tissues.

## Materials and methods

### Laboratory tests

All the orbital bone specimens without the periosteum layer were obtained from cadavers during a medicolegal autopsy performed no later than 2–5 days after death. All of them were victims of sudden deaths (*mors subita*) after accidents. The biological material was collected from 14 different cadavers between 20 and 51 years old (including 11 males and 3 females). The cadavers had not suffered from any chronic diseases or mechanical injuries to their heads according to the clinical interview. After opening the cranial cavity and collecting the brain for a routine histological examination, the orbital roof was exposed. After meninge removal, bone fragments were collected from the superior and medial walls, both from the left and right orbits. Each bone was cut alongside the sagittal plane (where the bone was straight) to obtain repeatable specimens that were 7–15 mm wide, 0.7–2.3 mm thick and 30–40 mm long. There was no possibility to test specimens cut in the longitudinal direction of the orbit due to its high curvature. Every specimen was individually measured and described and stored in a 0.9% saline solution (NaCl) at a temperature of − 20 °C for 6 to 36 h until the laboratory strength tests were performed. The above procedure was similar to the procedures performed by Waterhouse et al. and Morgan et al.^25,26^.

Finally, specimens were tested using a material testing machine (Zwick Roell Z020) equipped with a video-extensometer to obtain the Young’s modulus and the ultimate strength of the orbital bone in the longitudinal direction only, if possible, where the curvature of orbit was the lowest.

Every tested specimen was initially stretched by a force of *F*_0_ = 20 N with a velocity of *v*_0_ = 2 mm/s due to the elimination of the fixation inaccuracy between the grips and specimen. Then, they were finally stretched alongside the traverse direction of the testing machine with a velocity of *v* = 0.01 mm/s until the potential rupture of the specimen or until the potential slipping out of the specimen from the holding grips of the testing machine.

**Figure 1:**
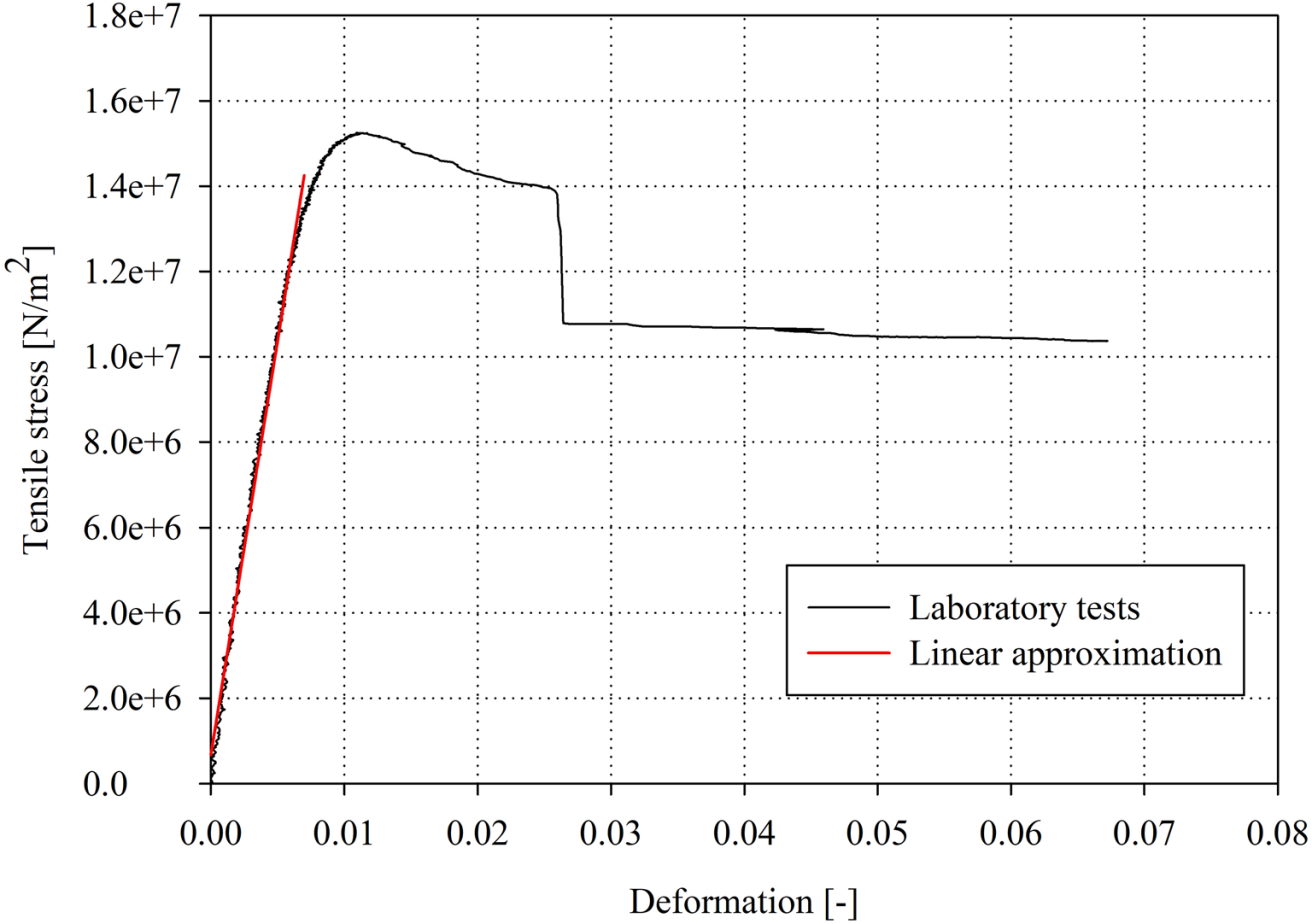
Function of the tensile stress—deformation of the laboratory test on exemplary orbital bone specimens (black) and the result of the linear approximation (red).

Afterwards, each specimen was cut at the middle of its length, and its cross-section area was calculated by the Autodesk AutoCAD program based on imported footprints of each cross-section *A* on paper with the preservation of the original scale. As during the experiments, only very small plastic effects were observed, and it was assumed that the cross areas before and after the test are the same. The tensile stress *σ* and strain *ε* were based on the data gathered from the test according to formulas (1) and (2):

1 $$\sigma = \frac{F}{A}$$

2 $$\varepsilon = \frac{\Delta l}{{l_{0} }}$$

where *F* is the value of the stretching force, *A* is the cross-sectional area of the tested specimen, *Δl* is the change in the video-extensometer gauge length, and *l*_*0*_ is the original gauge length. Afterwards, the tensile stress–strain function was prepared for each specimen using the Sigma Plot program. Then, the linear part of each function was selected, and using the Levenberg–Marquardt algorithm, linear regression was performed^27,28^. The pre-estimated parameters of the approximation were arbitrarily determined due to the linear approximation, which was unconditionally convergent (*a* = 1 and *b* = 0 for *y* = *a·x* + *b*). Such a calculated approximation of parameter *a* is the averaged Young’s modulus value sought (tangent of the angle between the linearly approximated function and the abscissa axis—see Fig. 1). Furthermore, the correlation coefficient *r* was determined for each tensile test. Note that consent for the research was obtained from the Bioethics Faculty Committee of Medical University of Gdansk as per the recommendations of the Helsinki Declaration^29^.

### Finite element model

The geometry of the finite element models of the human skull fragment comprising the orbit and its broad neighborhood (MOBE and MOBOSE) as well as the adjacent intraorbital structures (MOBOSE only) was built in AutoCAD software based on the skull geometry of a healthy male adult according to a computed tomography (CT) scan. As the contact of the abovementioned model parts was taken into account in the MOBOSE, each model part was created separately but matched one another. Mesh generation was executed in the MSC.Apex software, while further analysis was performed using the MSC.Marc/Mentat software (using dedicated solutions to avoid locking problems for incompressible and near incompressible responses^30^).

The considered model parts were composed of 3,616 triangular thin shell elements representing the bone structures, 5,828 tetrahedron solid elements representing the eyeball, 7,749 tetrahedron solid elements representing the generalized intraorbital soft tissues (composition of fat, muscles, veins and nerves), and 238 triangular thin shell elements representing the thin orbital septum (comprising the Whitnall and Lockwood ligament as well as canthal tendons and tarsal plates). The eyeball was modeled as a perfect homogeneous solid sphere based on the volume of a human eye. Because the most prominent feature of an eyeball is its incompressibility and the work focused on orbital bone damage, its mechanical properties were generalized according to eyeball layers based on the literature data, while the vitreous body was a dominant component. A similar approach was followed in the case of other intraorbital soft tissues that were combined as one homogeneous body between the eyeball and the orbit. However, the orbital septum was tied to the orbit having common nodes at its outer rim, while nodes from the inner rim of the septum were tied to the adjacent nodes of the eyeball by 38 nodal links. Notably, any joins within the bony part of the model were taken into account. The mechanical properties (all segments were assumed to be linear elastic isotropic materials) were individually assigned to the above-mentioned parts of the model based on the averaged data obtained from laboratory tests (orbital part only) as well as from the literature (see Table 1). To properly model the incompressible properties of the eyeball and the fat tissue, the Poisson’s ratio of *v* = 0.499999 was applied.

**Table 1:** Material properties applied to the FEM models of the human orbit region.

Part of the model	Density *ρ* (g/m^3^)	Poisson’s ration *ν* (–)	Young’s modulus *E* (N/m^2^)
Orbital bone^31,43^	1,610	0.33	1.3 × 10^9^
Skull bone^15,31,44^	1,800	0.33	1.3 × 10^10^
Eyeball^45,46^	1,000	0.499999	5.0 × 10^5^
Intraorbital tissues^45,47^	970	0.499999	1.0 × 10^4^
Orbital septum^48–50^	1,200	0.33	5.0 × 10^5^

**Figure 2:**
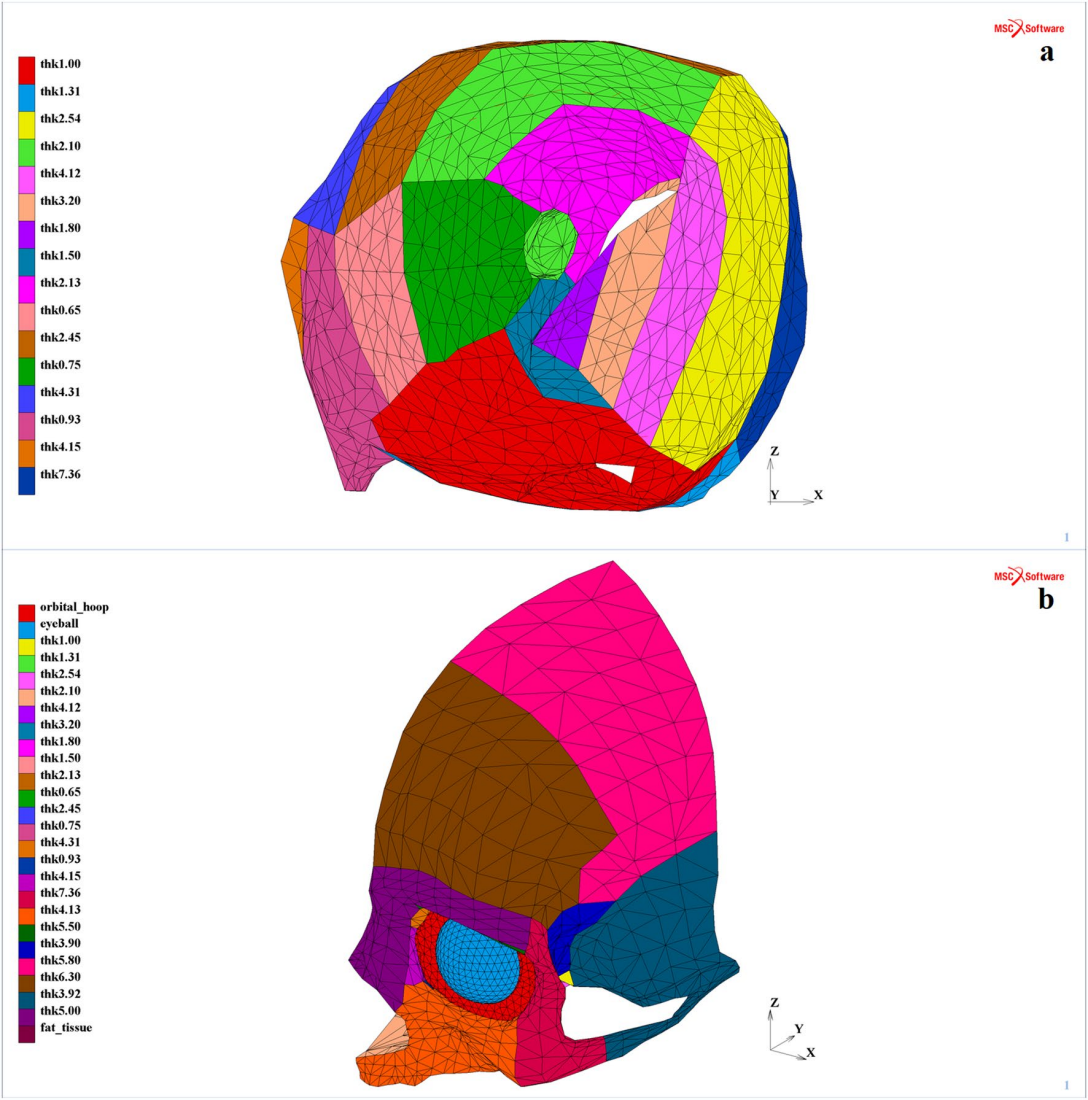
Thickness distribution in the model: (**a**) orbital part (frontal view) and (**b**) remaining part of the skull with intraorbital entities (isometric view)^30^.

The contact type (a node-to-segment variant) without friction was assumed. Then, three different contact bodies were established due to the contact problem implementation for the MOBOSE variant. They were, respectively, the eyeball, intraorbital soft tissues and the skull part model (including the orbit) combined with the orbital septum. Except for the abovementioned orbital septum, other contact bodies were not tied to one another. Afterwards, the geometry of the thin shell skull part of the model was diversified by applying individual thicknesses to the specified areas. The orbit thickness was detected based on the CT scan of healthy patients treated in the emergency unit with no traumatic history. Thus, the orbit was split into 16 regions with different thickness values within the range of 0.65–7.36 mm. Additionally, the remaining part of the considered skull fragment was divided into several regions, and the thickness distribution was executed according to the averaged values measured for the specific craniofacial bones present within the model, including maxillary, zygomatic, frontal, parietal, temporal and sphenoid bones (Fig. 2).

**Figure 3:**
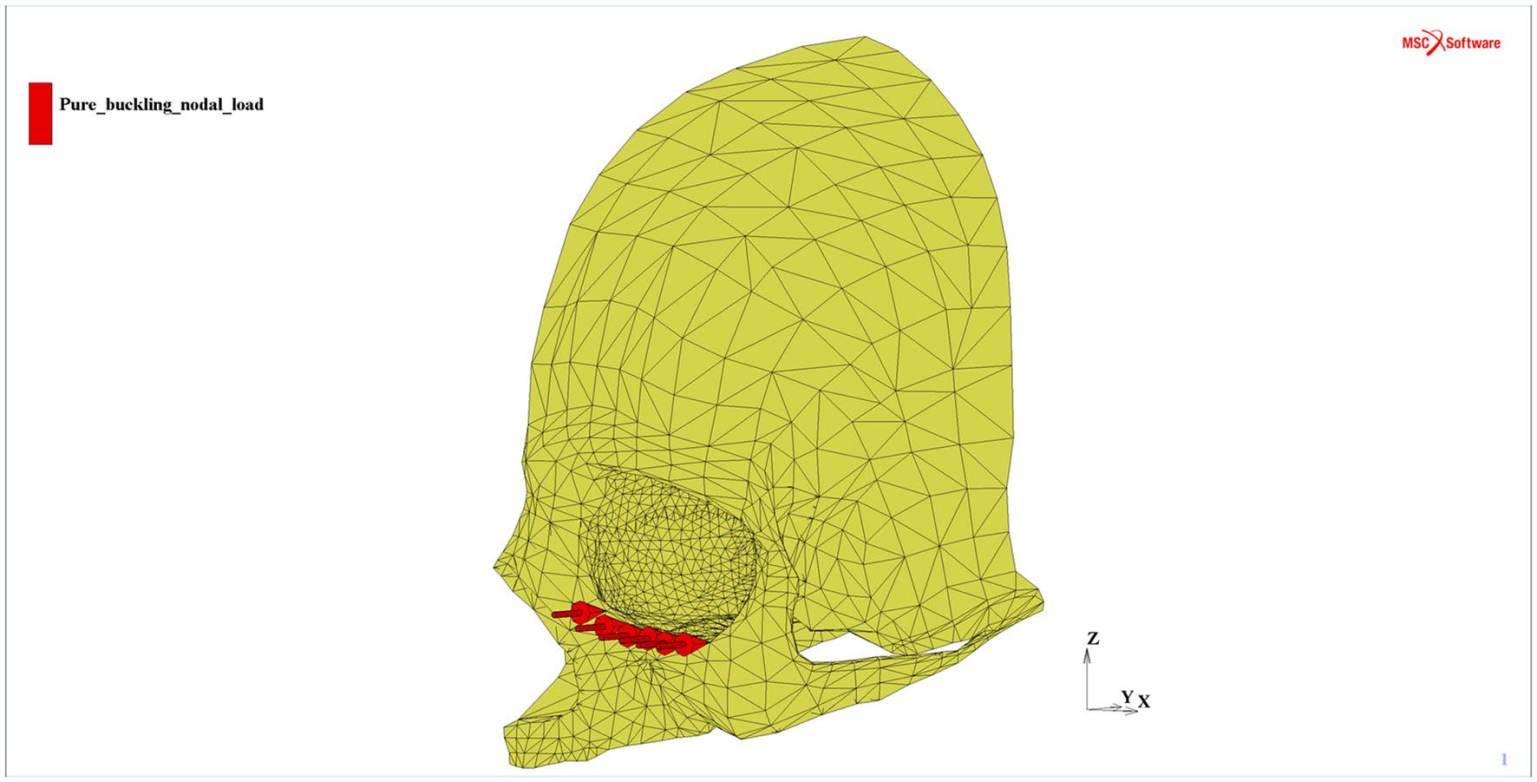
External load applied to the orbital lower rim—set of 6 nodal forces (red)^30^.

**Figure 4:**
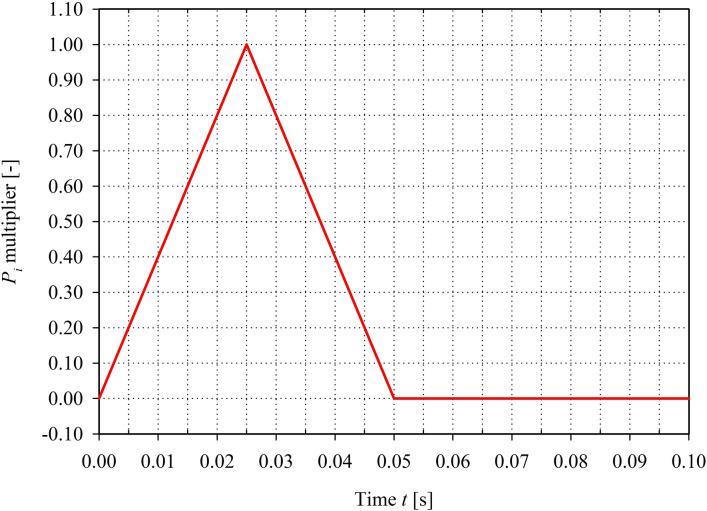
Time function of the load.

The fixed displacements in the *X, Y,* and *Z* directions were applied to all the nodes at the thin shell rim of the considered part of the skull where the model was to be connected to the remaining part of the complete human skull. The selected part of the skull was large enough to avoid the effect of the boundary conditions influence on the stress distribution in the orbital region. In contrast to the above boundaries, the external load of the pure “buckling” mechanism impact was directly applied to the orbital lower rim in the global Y direction within the Frankfurt plane model as a set of 6 equal nodal forces of 2,400 N (Fig. 3), giving the sum of 14,400 N^10^. The total value applicable for all analyzed load cases corresponded to the doubled equivalent of the energy of the impact destructive tests reported by Schaller et al.^31^. The proposed load value was selected to cause damage for the case of the “buckling” mechanism of blow-out fracture, as the load was applied directly to the orbital rim that had higher stiffness than the orbital bone. Furthermore, the load was applied according to the specified time function (Fig. 4), which was valid for all the considered models (MOBE and MOBOSE). The impulse corresponded to the averaged time of the impact of a car frontal airbag in contact with the craniofacial part of a skull during a car accident^32,33^.

Prior to the transient analysis, the first two natural frequencies of the thin shell skull structure finite model (including the orbit) were calculated as follows to implement the damping effect considered in the skull model and represent an existing physical phenomenon:

3 $$\begin{aligned} & \omega_{1} = 2.4438 \cdot 10^{3} \, [1/s] \\ & \omega_{2} = 3.7262 \cdot 10^{3} \, [1/s] \\ \end{aligned}$$

To obtain the damping matrix **C**, the Rayleigh approach was applied:

4 $${\mathbf{C}} = \alpha {\mathbf{M}} + \beta {\mathbf{K}}$$

where **M** is the mass matrix and **K** is the stiffness matrix. It is necessary to determine the Rayleigh’s damping coefficients (*α* and *β*)^34^:

5 $$\alpha = \frac{{2\omega_{1} \omega_{2} \xi }}{{\omega_{1} + \omega_{2} }};\quad \beta = \frac{2\xi }{{\omega_{1} + \omega_{2} }}$$

where *ξ* is the logarithmic decrement. The logarithmic decrement *ξ* = 0.053 was applied to the model as per the work by Huang et al.^35^. The calculated values according to formula (5) are given as follows:

6 $$\alpha = 1.5644 \cdot 10^{3} ;\quad \beta = 1.7180 \cdot 10^{ - 5}$$

Finally, the nonlinear transient dynamic analysis regarding the contact problem according to the algorithm proposed by Houbolt with the constant integration step of Δ*t* = 1.0 × 10^–4^ s was performed for two different models: the bony part of the skull exclusively and the bony part of the skull including the adjacent intraorbital soft tissues^36^.

### Ethics approval and consent to participate

The authors obtained consent from the Independent Bioethics Committee for the research, including:

- the use of biological materials (tissue samples),
- the use of imaging tests (CT scans—after anonymization),
- the use of information from medical records (after anonymization).

The consent was in accordance with the European Union’s Directive 2004/23/WE art. 13,15^51^. The authors also declare that they complied with the Helsinki Declaration (World Medical Association, Fortaleza, Brazil, 2013)^29^.

Informed consent was obtained from the interviewers for the use of clinical data and accompanying images, when possible.

## Results

### Laboratory test outcomes

The outcomes of the laboratory tests as well as the averaged value of the Young’s modulus calculated for the orbital bone and the standard deviation for the whole group are summarized in Table 2. Moreover, the specimens were distinguished by the collection location from the orbit. For those taken from the superior wall, the average calculated Young’s modulus was *E*_*S*_ = 1.29 GPa, with a mean correlation coefficient *r*_*iS*_ = 0.9802. The calculated standard deviation for the superior wall was *SD*_*S*_ = 1.50. The calculated average Young’s modulus for the medial wall was *E*_*M *_= 1.36 GPa, and the corresponding mean correlation coefficient was *r*_*iM *_= 0.9920. The standard deviation calculated for the medial wall was *SD*_*M*_ = 0.618.

**Table 2:** Young’s moduli values of the whole group.

Specimen no	Specimen’s origin (orbital wall)	Gender	Age	Young’s modulus *E*_*i*_ (Pa)	Correlation coefficient *r*_*i*_ (–)
1	Superior	Female	45	1.19 × 10^8^	0.9927
2	Superior	Female	45	4.73 × 10^8^	0.9971
3	Superior	Male	38	1.06 × 10^9^	0.9601
4	Superior	Male	51	5.49 × 10^8^	0.9829
5	Superior	Male	51	5.50 × 10^8^	0.9858
6	Superior	Male	39	3.82 × 10^8^	0.9983
7	Superior	Male	39	3.47 × 10^8^	0.9967
8	Medial	Male	53	1.60 × 10^8^	0.9981
9	Superior	Female	43	5.85 × 10^8^	0.9888
10	Superior	Female	43	5.91 × 10^8^	0.9890
11	Superior	Female	43	5.85 × 10^8^	0.9888
12	Superior	Male	49	7.81 × 10^8^	0.9917
13	Medial	Male	50	1.62 × 10^9^	0.9914
14	Superior	Male	50	1.15 × 10^9^	0.9921
15	Superior	Male	47	9.43 × 10^8^	0.9996
16	Medial	Male	47	1.50 × 10^9^	0.9910
17	Superior	Male	53	6.36 × 10^8^	0.9948
18	Superior	Female	32	2.53 × 10^9^	0.9905
19	Medial	Male	46	1.94 × 10^9^	0.9957
20	Superior	Male	46	8.26 × 10^8^	0.9962
21	Superior	Male	46	1.86 × 10^9^	0.9822
22	Superior	Male	46	2.91 × 10^9^	0.9486
23	Medial	Male	20	1.56 × 10^9^	0.9837
24	Superior	Male	20	1.95 × 10^9^	0.9118
25	Superior	Male	43	7.20 × 10^9^	0.9106
26	Superior	Male	43	1.02 × 10^9^	0.9859
Average		n/a	43	1.30 × 10^9^	0.9825
Standard deviation		n/a	6.59	1.38 × 10^9^	n/a

The specimens were of an irregular shape; therefore, it was difficult to fix them properly in grips without causing damage during grip clamping. When a relatively high force was applied during the tests, the slip-out effect was observed for almost all the specimens. This was the reason why it became impossible to determine either the ultimate stress value or the yield limit of the orbital bone. Therefore, the mean yield criterion of a Huber–Mises–Hencky (H–M–H) stress (also known as the von Mises equivalent stress) of 150 MPa was assumed (obtained during the strength test on bone pieces by Nagasao et al.^37^). The same value was assumed as the ultimate stress and the fracture criterion for the orbital bone.

### Finite element analysis

**Figure 5:**
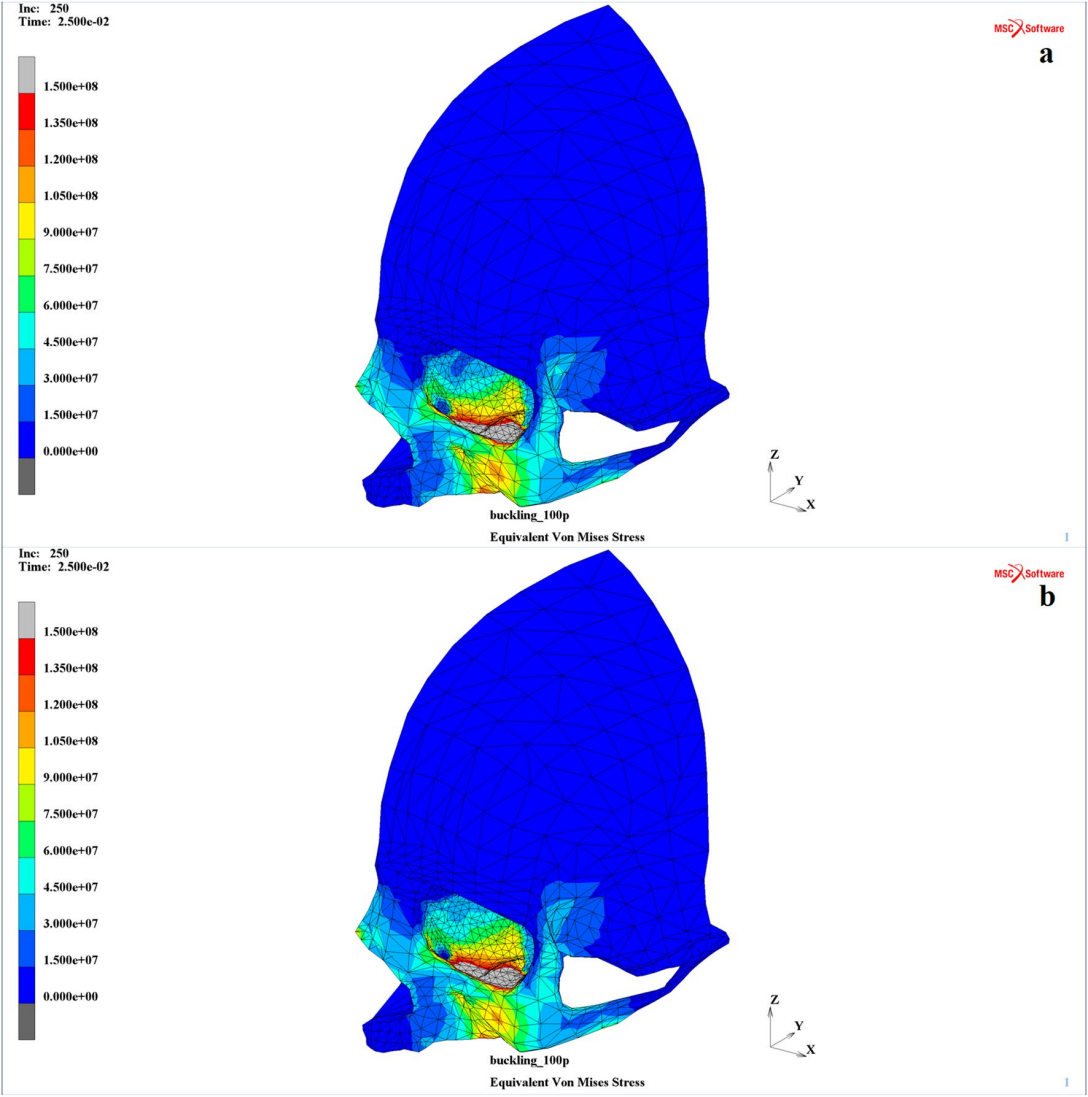
H–M–H stress (Pa) distribution within the analyzed thin shell skull part during the time t = 25 ms corresponding to the extremal value of the external load: (**a**) MOBE and (**b**) MOBOSE^30^.

Despite the structural differences between the two investigated models of a human skull during the nonlinear transient analysis, the observed areas where the H–M–H equivalent stress exceeded the assumed ultimate stress value, which was considered to be the potential damage area during the application of the mentioned total external load, seemed to be similar for both the MOBE and MOBOSE cases (Fig. 5). Almost all of the anticipated damage in both models was concentrated at the lower wall, just behind the orbital lower rim, including the maxilla and the zygomatic bones only; the second, smaller independent damage also occurred in both analyzed cases within the lacrimal bone, right between the lacrimal fossa and the orbital rim. However, three fundamental differences between the outcomes obtained for those two models were observed.

The first difference was the difference in the H–M–H stress distribution within the bony parts of the two models. When the “buckling” mechanism in the MOBE was considered, the stress propagation was far beyond the orbit and the area of the external load application, i.e., the lower orbital rim. Noticeable values of the H–M–H stress were observed not only at a larger area within the orbit and the maxillary part of the orbital rim compared to the second model regarding the contact problem (MOBOSE) but also at the upper orbital rim and the temple, involving substantial parts of the frontal and temporal bones. Furthermore, the analysis revealed that the orbital wall might not be the only area endangered by bone damage under the applied maximal load value during the dry skull analysis. Surprisingly, a slight exceedance of the applied ultimate stress value limit was also observed within the infraorbital foramen. However, other concentration areas within the bony part of the model were observed in the dry skull case, which were especially visible at the lower part of the maxilla or the nasal bone; nevertheless, they should be seen as local concentrations caused by adjacent hinged nodal boundary conditions. On the other hand, the H–M–H stress distribution during the analysis of the contact model regarding the intraorbital soft tissues was smoother compared to the dry skull case. The noticeable areas of the H–M–H stresses were limited mainly to the orbit as well as the maxilla and zygomatic bones, except the local effects caused by the boundary conditions occurred on the outskirts of the model.

The second characteristic difference was the time of the potential damage occurrence within the orbit. When analyzing the MOBE, the very first symptom of orbital floor breakage was observed 1.5 ms earlier than for the same impulse applied to the model concerning the presence of intraorbital soft tissues (MOBOSE). That difference implies the external destructive load value corresponding to the time of the damage occurrence within the orbit. The load necessary to cause damage was over 11% higher in the intraorbital contact model than it had been observed for the MOBE analysis. Accurate data for those two analyzed models regarding the times of the first damage occurrence and the corresponding destructive load values are summarized in Table 3.

**Table 3:** Summary of the analysis.

Model type	Time of the first fracture occurrence within the orbit (ms)	External load value corresponding to the first fracture occurrence (N)	Potential fracture area during the maximal load (mm^2^)
Bones only model (MOBE)	13.3	7,660	270
Contact model (MOBOSE)	14.8	8,520	215

The third significant difference is the observation that a visible part of the impact burden for the MOBOSE was shifted from the lateral part of the maxilla bone towards its medial part within the orbital floor compared to the MOBE. Moreover, the anticipated damage area was approximately 20% lower during the analysis of the extended model of the skull, including the contact problem between the intraorbital soft tissues and the bony orbit, than in the case of the dry skull pure “buckling” mechanism. The detailed data on the estimated damage areas based on the H–M–H extreme stress maps for both analyzed models corresponding to the time of the maximal external load are also summarized in Table 3.

**Figure 6:**
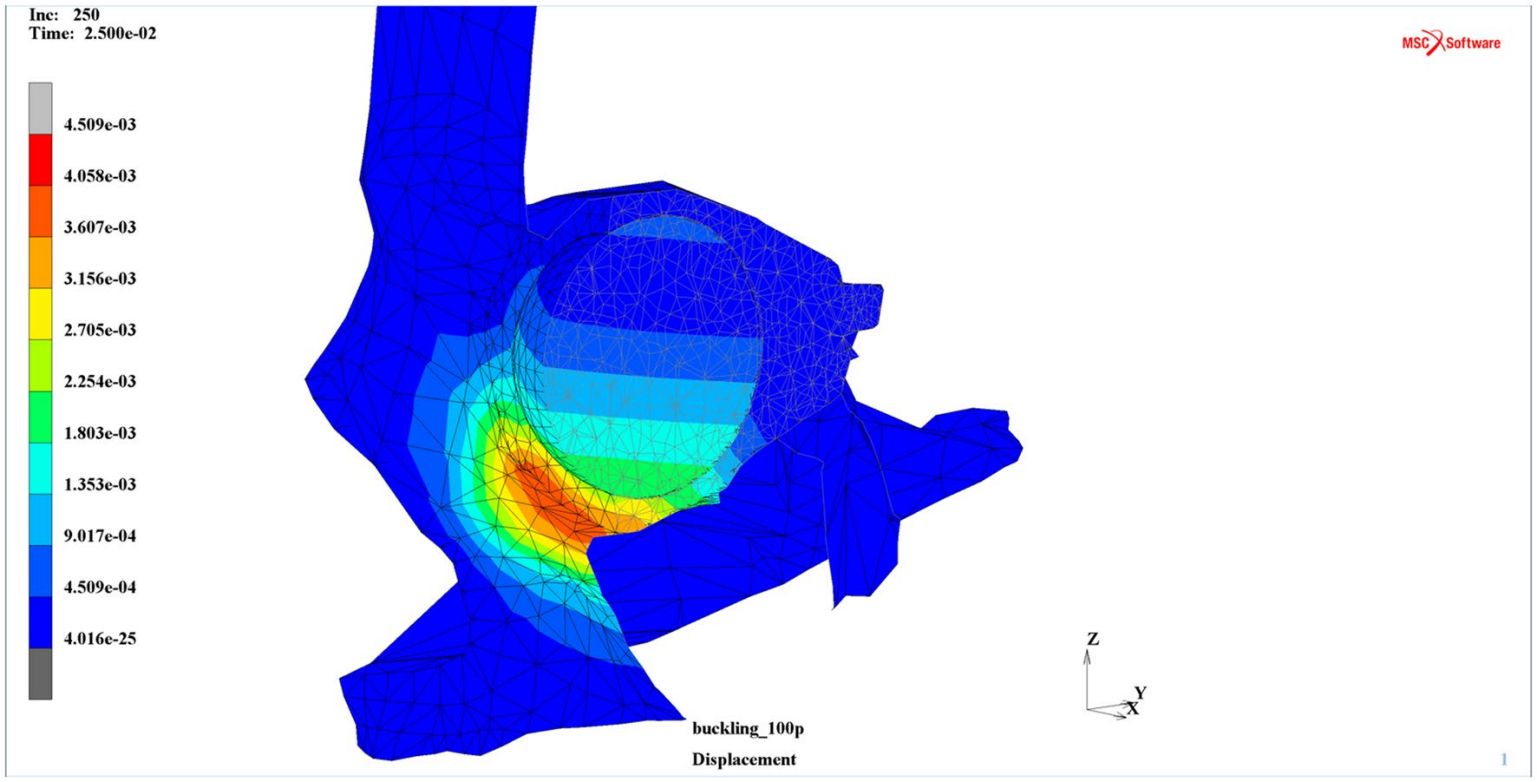
Displacements map (m) with the deformation for the vertical cross-section in the contact model including intraorbital soft tissues (t = 25 ms)^30^.

Additionally, it is worth mentioning that the displacements of the intraorbital soft tissues were substantial compared to the displacements of the orbit and its rim, even though the intraorbital soft tissues were not directly affected by the external dynamic load at all. However, the displacement propagation within the intraorbital entities may be seen as a result of a contact problem assigned between the orbital and intraorbital parts of the model (Fig. 6).

In contrast to other authors’ observations (except Foletti et al.^20^), the energy of the impact causing orbital wall damage reported in the current study was substantially higher: 15.5 J and 16.2 J for the MOBE and the MOBOSE, respectively. The value of the energy for the pure “buckling” mechanism calculated by Nagasao et al.^37^ was 0.857 J, based on the theoretical formulas proposed by Warwar et al.^38^. The analogous value of the energy indicating orbital wall damage was reported by Ahmad et al., who conducted laboratory tests by dropping an impactor with a mass of 232 g onto the orbital rim of cadavers from a height of 0.675 m, which was 1.54 J^39^. On the other hand, Foletti et al. reported an extremal kinetic energy of up to 12.25 J, which was the same order of magnitude as in the current work^20^.

### CT scan analysis: validation of the numerical model

Finally, the results obtained during the numerical analysis were compared with the CT scans of 23 patients (5 females, 17 males between ages 19 and 81, with a mean age of 41) suffering from orbital wall injuries that might be classified as the pure “buckling” mechanism according to the clinical history of those patients available in the University Clinical Centre in Gdańsk. In all cases, fractures were located at the lower orbital wall, both with and without the displacement of the bone fragments to the adjacent maxillary sinuses. The scale of the observed fractures varied depending on the real striking force, which was unknown according to the medical statistics; however, the numerical simulations aimed to estimate the potential energy of the impact, providing that the impact was distributed to the lower orbital rim solely in the Frankfort plane. The radiological assessment was independent of the modeling. The current FEM model was used to compare to the selected CT scans and showed that the numerical simulation outcomes were congruent with the clinical images of patients classified as “buckling” blow-out fractures cases. Both the localization and the range of the potential damage were similar to the clinical observations. Nevertheless, the size of those fractures was not evaluated due to the lack of precise enough data for each case (exact direction and area of impact).

Hence, it was pointless to evaluate the precise area of the fracture for those numerical simulations. Moreover, using the linear elastic isotropic model of the material allowed, at most, the potential fractures to be estimated, but their exact size could not be determined precisely.

**Figure 7:**
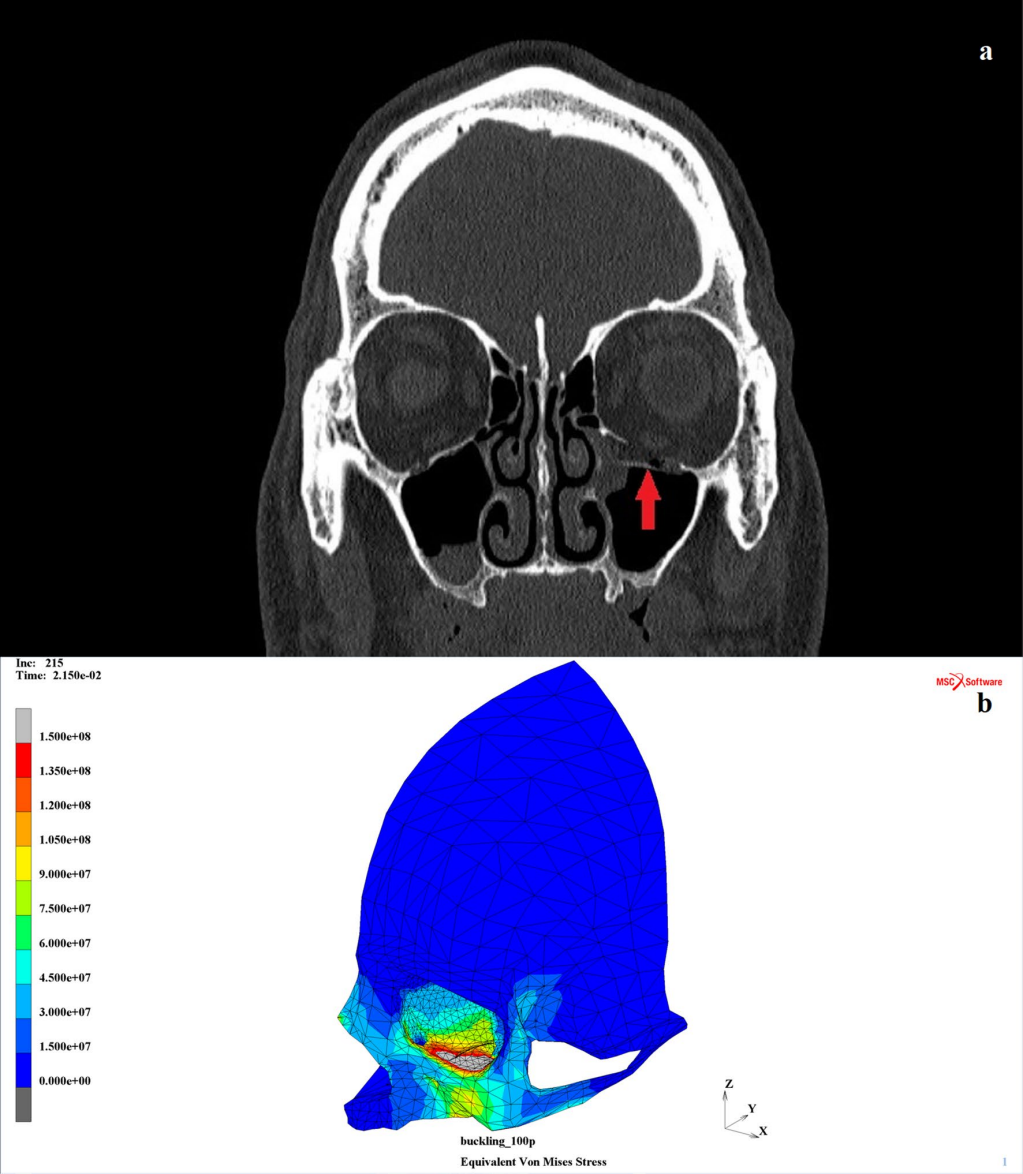
CT scan analysis and the impacting load assessment: (**a**) exemplary patient (male, 48) with substantial-scale fracture of the lower wall of the left orbit (red arrow) and (**b**) corresponding scale of the orbital damage for the time t = 21.5 ms in the MOBOSE^30^.

According to the CT scan of an exemplary patient selected for the analysis, a substantial-scale blow-out fracture within the lower wall of the left orbit was found (Fig. 7a). The analyzed injury occurred as a result of a traffic accident, and the short-term loss of consciousness of the patient was also reported. According to the numerical simulations, the anticipated load might have exceeded the value of 12,000 N there, as the similar scale of the potential orbital damage in the MOBOSE analysis for the time *t* = 21.5 was observed (Fig. 7b).

## Discussion

### Laboratory part

Laboratory tests for various types of bones were performed by other authors in previous works^26,40,41^, but most of them were concerned with bone specimens with uncomplicated geometry, such as long or flat bones. Moreover, another great work regarding the human craniofacial material parameters held by Dechow et al.^42^ also deserves attention. Dechow et al. successfully determined the material parameters such as the density, Young’s modulus, shear modulus, and Poisson’s ratio in three directions using the ultrasonic wave technique within the circumorbital and other craniofacial bones. A comprehensive study of orbital bones is still missing in the literature, especially in the context of tensile strength tests. In fact, those bones are completely different from other bones, as they are relatively thin, fragile and have a complex curvature. This implies that it is necessary to take an individual approach. To avoid the main obstacle resulting from the complex curvature of the human orbit, the authors decided to take specimens in the sagittal plane only. That method of sampling improved the quality of the test, but unfortunately, it was still not enough to fix the specimens in the testing machine’s grips, which could finally allow us to identify the yield limit. Even though the tensile strength test held upon orbital bone specimens was unique to the authors’ best knowledge, it still needs some improvements, especially in terms of the fixation of the specific samples in the testing machine. Unfortunately, for the biological material, it is impossible to expose specimens too long before testing; thus, it was impossible to wait until the glue or cement became dry between the specimen and any other auxiliary material, which was also taken into consideration as a possible method. Therefore, the authors are still making efforts to find a way to solve this problem by a completely different technique. After commencing the tensile strength tests as well as orbital bone sampling process, the Authors suppose that orbital bone may not have orthotropic properties, which is what was postulated by other authors. The orbital wall neither has any orthogonality within its structure nor has any other fundamental differences in it but a variable thickness. Hence, the isotropic parameters applied to the current simulation do not necessarily produce a simplified model of the material. Nevertheless, further tests will be conducted to investigate whether any noticeable difference in the Young’s modulus between specimens sampled in the sagittal and coronal planes of the orbit is observable.

### Numerical part

The “buckling” mechanism may be seen as the most frequently numerically tested mechanism among other orbital blow-out trauma mechanisms; however, the current analysis revealed some interesting observations^10^. The most substantial difference was the noticeable difference in the destructive load value between the two analyzed models: the bones-only model (MOBE) and the system of contact bodies including the intraorbital soft tissues (MOBOSE). In spite of the fact that the impulse was applied to the stiffest part of the model (i.e., the orbital rim), the presence of the intraorbital entities helped to mitigate the propagation of the impact through the bony part of the skull, thus increasing the threshold of the external load necessary to cause damage to the orbital wall. Hence, the anticipated damage area for the given excessive external load was also visibly smaller when taking the intraorbital tissues into consideration. Notably, the performed validation showed a relatively high convergence between the numeric simulations and the real clinical cases based on the CT scan analysis of patients suffering blow-out traumas identified as the “buckling” mechanism according to the clinical interview. In particular, the work showed that it is possible to assess the load value that could cause orbital wall fracture as well as the direction of the impact based on the actual size and the location of the fracture, according to the CT scan. It could also help in developing personal protection equipment.

The presence of intraorbital soft tissues plays a substantial role in the model performance. They not only protect the access of the potential impactor to the deeper parts of the orbit but also increase the vibration resistance of the complex model of the orbital region. The observed vibrations within the orbit in the MOBOSE had visibly lower amplitudes than the analogous values in the MOBE. It should be noted that any additional damping was applied to those soft tissues. The mentioned effect was achieved only using the nonlinear variant of the recurrent Houbolt algorithm that owed its effectiveness due to the built-in strong numerical dissipation effect. Undeniably, the intraorbital soft tissues helps dampen the vibration, thus facilitating the convergence of the solution, so the determination of the actual damping effect within the intraorbital tissues seems to be an interesting direction of further research. Nevertheless, the most important factor that influenced the increase in the impact resistance was the deformation of the intraorbital soft tissues. Although the load did not affect the intraorbital entities directly, the deformation of the bony part of the model implied the deformation of those intraorbital structures. Through their deformation, some portion of the kinetic energy of the impact was engaged, thus relieving the bony part of the orbit. Moreover, it may be presumed that the deformation of the intraorbital bodies may be one of the most important factors through other complex blow-out mechanisms, especially when applying the external load to the orbit indirectly via the intraorbital soft bodies.

According to the authors’ best knowledge, this was the first successfully conducted attempt to engage the impacting dynamic load as a set of nodal forces applied to the orbital system regarding the contact problem between intraorbital tissues and the orbit using the FEM. All other works so far focused on blunt impact simulations.

The analysis has shown the importance of taking intraorbital soft tissues into consideration when analyzing blow-out fracture mechanisms. An apparently obvious case of the pure “buckling” mechanism was proven to be considerably dependent on the presence of the intraorbital entities, which causes a substantial change in the distribution of stress through the model. The role of the intraorbital tissues is to absorb a considerable portion of the kinetic energy of the impact and to transform it into deformation. The change in the shape of the relatively soft body mitigates shock wave propagation within the bone structure of the orbit. Moreover, the presence of intraorbital entities limits the freedom of the deformation of bone structures to a certain extent due to their incompressibility.

Further work is planned to investigate the impact of the presence of intraorbital soft tissues through other more complex orbital blow-out mechanisms.

## Data Availability

The datasets generated during and/or analyzed during the current study are available from the corresponding author on reasonable request.

## References

[1] Clarke PRR (1971). Fractures of the orbit: (Proceedings of the symposium on orbital fractures, 19–20 April, 1969, Amsterdam). J. Neurol. Sci..

[2] Jha K, Rajalakshmi A (2018). Evaluation and management of orbital trauma. J. Clin. Ophthalmol. Res..

[3] Paluch J, Markowski J (2013). Interdisciplinary surgical management of orbital and maxillo-ethmoidal complex disorders. Clinical Management and Evolving Novel Therapeutic Strategies for Patients with Brain Tumors.

[4] Weinzweig J, Taub PJ, Bartlett SP, Weinzweig J (2010). Fractures of the orbit. Plastic Surgery Secrets Plus.

[5] Bregstein J, Roskind CG, Sonnett FM, Polin RA, Ditmar MF (2011). Emergency medicine. Pediatric Secrets.

[6] Hogg NJV, Stewart TC, Armstrong JEA, Girotti MJ (2000). Epidemiology of maxillofacial injuries at trauma hospitals in Ontario, Canada, between 1992 and 1997. J. Trauma Acute Care Surg..

[7] Shin JW, Lim JS, Yoo G, Byeon JH (2013). An analysis of pure blowout fractures and associated ocular symptoms. J. Craniofac. Surg..

[8] Einy S, Abdel Rahman N, Siman-Tov M, Aizenbud D, Peleg K (2016). Maxillofacial trauma following road accidents and falls. J. Craniofac. Surg..

[9] Converse JM, Smith B (1950). Reconstruction of the floor of the orbit by bone grafts. Arch. Ophthalmol..

[10] Smith B, Regan WF (1957). Blow-out fracture of the orbit: mechanism and correction of internal orbital fracture. Am. J. Ophthalmol..

[11] Bandyopadhyay TK, Sapru BL (2004). Management of an isolated orbital blow-out fracture. Med. J. Armed Forces India.

[12] Felding UNA (2018). Blowout fractures—clinic, imaging and applied anatomy of the orbit. Dan. Med. J..

[13] Fujino T (1974). Experimental, “blowout” fracture of the orbit. Plast. Reconstr. Surg..

[14] Fujino T, Makino K (1980). Entrapment mechanism and ocular injury in orbital blowout fracture. Plast. Reconstr. Surg..

[15] Nagasao T (2006). The effect of striking angle on the buckling mechanism in blowout fracture. Plast. Reconstr. Surg..

[16] Al-Sukhun J, Kontio R, Lindqvist C (2006). Orbital stress analysis—part I: simulation of orbital deformation following blunt injury by finite element analysis method. J. Oral Maxillofac. Surg..

[17] Ross CF (2011). In vivo bone strain and finite-element modeling of the craniofacial haft in catarrhine primates. J. Anat..

[18] Al-Sukhun J, Penttilä H, Ashammakhi N (2012). Orbital stress analysis, part V: systematic approach to validate a finite element model of a human orbit. J. Craniofac. Surg..

[19] Patel S, Andrecovich C, Silverman M, Zhang L, Shkoukani M (2017). Biomechanic factors associated with orbital floor fractures. JAMA Facial Plast. Surg..

[20] Foletti JM (2019). Development and validation of an optimized finite element model of the human orbit. J. Stomatol. Oral Maxillofac. Surg..

[21] Foletti JM (2019). Finite element analysis of the human orbit. Behavior of titanium mesh for orbital floor reconstruction in case of trauma recurrence. J. Stomatol. Oral Maxillofac. Surg..

[22] Skorek A (2013). Dynamic Analysis of Orbital Blow-Out Injury Based on Numerical Model of the Orbit and Clinical Observations.

[23] Kłosowski P, Skorek A, Zmuda Trzebiatowski M, Pietraszkiewicz W, Gorski J (2014). Static and dynamic modelling blow-out type of trauma of orbital wall. Shell Structures: Theory and Applications.

[24] Skorek A (2014). Posttraumatic orbital emphysema: a numerical model. J. Ophthalmol..

[25] Waterhouse N, Lyne J, Urdang M, Garey L (1999). An investigation into the mechanism of orbital blowout fractures. Br. J. Plast. Surg..

[26] Morgan EF, Bayraktar HH, Keaveny TM (2003). Trabecular bone modulus-density relationships depend on anatomic site. J. Biomech..

[27] Levenberg K (1944). A method for the solution of certain non-linear problems in least squares. Q. Appl. Math..

[28] Marquardt DW (1963). An algorithm for least-squares estimation of nonlinear parameters. J. Soc. Ind. Appl. Math..

[29] World Medical Association (2013). World Medical Association Declaration of Helsinki: ethical principles for medical research involving human subjectsworld medical association declaration of helsinkispecial communication. JAMA.

[30] Microsoft Corporation, MSC. Marc/Mentat Manual (2019).

[31] Schaller A, Huempfner-Hierl H, Hemprich A, Hierl T (2013). Biomechanical mechanisms of orbital wall fractures—a transient finite element analysis. J. Cranio-Maxillofac. Surg..

[32] Rosenbluth W (2001). Investigation and Interpretation of Black Box Data in Automobiles: A Guide to the Concepts and Formats of Computer Data in Vehicle Safety and Control Systems.

[33] Nordhoff LSJ (2005). Motor Vehicle Collision Injuries: Biomechanics, Diagnosis, and Management.

[34] Strutt JW (2011). The Theory of Sound. Cambridge Library Collection—Physical Sciences.

[35] Huang BW, Kung HK, Chang KY, Hsu PK, Tseng JG (2009). Human cranium dynamic analysis. Life Sci. J..

[36] Houbolt JC (1950). A recurrence matrix solution for the dynamic response of elastic aircraft. J. Aeronaut. Sci..

[37] Nagasao T, Miyamoto J, Shimizu Y, Jiang H, Nakajima T (2010). What happens between pure hydraulic and buckling mechanisms of blowout fractures?. J. Cranio-Maxillofac. Surg..

[38] Warwar RE, Bullock JD, Ballal DR, Ballal RD (2000). Mechanisms of orbital floor fractures: a clinical, experimental, and theoretical study. Ophthalmic Plast. Reconstr. Surg..

[39] Ahmad F, Kirkpatrick NA, Lyne J, Urdang M, Waterhouse N (2006). Buckling and hydraulic mechanisms in orbital blowout fractures: fact or fiction?. J. Craniofac. Surg..

[40] Pal S, Pal S (2014). Mechanical properties of biological materials. Design of Artificial Human Joints and Organs.

[41] Seong W-J (2009). Elastic properties and apparent density of human edentulous maxilla and mandible. Int. J. Oral Maxillofac. Surg..

[42] Dechow PC, Wang Q, Peterson J (2010). Edentulation alters material properties of cortical bone in the human craniofacial skeleton: functional implications for craniofacial structure in primate evolution. Anat. Rec..

[43] Robbins DH, Wood JL (1969). Determination of mechanical properties of the bones of the skull. Exp. Mech..

[44] Peterson J, Dechow PC (2003). Material properties of the human cranial vault and zygoma. Anat. Rec. Part A Discov. Mol. Cell. Evol. Biol..

[45] Schutte S, van den Bedem SPW, van Keulen F, van der Helm FCT, Simonsz HJ (2006). A finite-element analysis model of orbital biomechanics. Vis. Res..

[46] Duck FA, Duck FA (1990). Chapter 4—acoustic properties of tissue at ultrasonic frequencies. Physical Properties of Tissues.

[47] Schoemaker I (2006). Elasticity, viscosity, and deformation of orbital fat. Investig. Ophthalmol. Vis. Sci..

[48] McKee CT, Last JA, Russell P, Murphy CJ (2011). Indentation versus tensile measurements of Young’s modulus for soft biological tissues. Tissue Eng. Part B Rev..

[49] Mukherjee S, Chawla A, Karthikeyan B (2006). A review of the mechanical properties of human body soft tissues in the head, neck and spine. Inst. Eng. J..

[50] Roberts JC (2007). Computational and experimental models of the human torso for non-penetrating ballistic impact. J. Biomech..

[51] The European Parliament and the Council of the European Union (2004). Directive 2004/23/EC of 31 March 2004 on setting standards of quality and safety for the donation, procurement, testing, processing, preservation, storage and distribution of human tissues and cells. Off. J. Eur. Union.

